# Characteristics of catechin loading rice porous starch/chitosan functional microsphere and its adsorption towards Pb^2+^

**DOI:** 10.1016/j.heliyon.2022.e10048

**Published:** 2022-07-30

**Authors:** Suwei Jiang, Hailiang Hu

**Affiliations:** a Department of Biological and Environmental Engineering, Hefei University, Hefei 230601, Anhui, China; b Department of Blood Transfusion, The First Affiliated Hospital of Anhui Medical University, Anhui, 230022, China

**Keywords:** Polyphenol, Adsorption kinetics, Composite microspheres

## Abstract

In this paper, we explore the adsorption potential of catechin (CT) loaded composite microspheres and provide a new micron scale carrier of functional factor. Chitosan (CS) modified rice porous starch (RPS/CS) was used as a CT adsorption carrier to prepare bioactive CT-loaded composite microspheres (CT@RPS/CS). The adsorption kinetics, storage characteristics, and biological activity maintenance of CT@RPS/CS were studied in an aqueous solution, and the sustained-release characteristics of CT@RPS/CS were studied *in vitro* during simulated gastrointestinal digestion. An aqueous solution further studied the removal characteristics of adsorbed heavy metal ion Pb^2+^. RPS/CS can significantly improve the ability to adsorb CT. RPS/CS can also significantly improve CT's storage stability, antioxidant stress, and slow-release characteristics, and the sustained release effect in gastric and intestinal juice. CT@RPS/CS can be removed Pb^2+^ by adsorbing in the solution, and their adsorption was physical adsorption and chemisorption, but the primary interaction is chemisorption. CT@RPS/CS can be used as a micron carrier of new food functional factors, which has potential space for improving and expanding the functional characteristics of its loaded functional factors and the endowing of new functions.

## 1 Introduction

Polyphenols are prone to moisture absorption, browning, oxidation, and lose biological function when exposed to the natural environment. Catechin (CT) is the main component of tea polyphenols. Many studies have shown that CT has a significant effect on antioxidant, anti-cancer [1, 2], antibacterial [3, 4], and anti-inflammatory abilities [5, 6], and CT can reduce lipid production and reduce the incidence rate of cardiovascular and cerebrovascular diseases [7, 8], and which can effectively improve the body's immunity, and protect the heavy metals poisoning. It has been widely applicated in food, medicine, and other fields. However, the active phenolic hydroxyl groups make CT more sensitive to environmental conditions (such as light, high temperature, humidity, and alkalinity). They are easy to oxidative browning, resulting in the decline or disappearance of activity and declined bioavailability [9].

Chitosan (CS) is rich in many sources, mainly extracted from chitin and prepared by deacetylation. The CS has biological function, non-toxicity, biodegradability, and good biocompatibility. Chitosan can remove DPPH, hydroxyl, and superoxide free radicals [10, 11] and show antioxidant capacity. The amino and hydroxyl groups of chitosan can form intramolecular and intermolecular hydrogen bonds to form a three-dimensional network structure, which can chelate heavy metal ions and exhibit adsorption properties [12, 13]. However, it is difficult for chitosan to release catechin, which affects its antioxidant effect.

At present, the methods of removing lead from solution include adsorption [14], ion exchange [15], membrane filtration [16], electrodialysis [17] and biological treatment [18, 19]. Previous studies [20] have shown that porous microspheres have very high adsorption efficiency for Pb2+. Researchers can modify the material to obtain higher adsorption capacity [21, 22], and some materials also have the characteristics of reuse [23]. Studies [24] demonstrated that modified starch could remove Pb^2+^ from industrial wastewater. Compared with other cereal starch, nature rice starch (NRS) has a small particle size, uniform size, and high specific surface area. It can be widely used in papermaking, textile, medicine, chemical industry. Rice porous starch (RPS) is generally prepared from rice starch by enzyme method, chemical method, or combination of chemical method and enzyme method.

Our previous study found that RPS/CS composite functional microsphere had good adsorption characteristics, sustained release characteristics, and storage stability for proanthocyanidin [24]. RPS/CS composite functional microsphere carrier has the potential advantage of loading catechin, but there is no relevant literature. This paper mainly discusses that catechin loading rice porous starch/chitosan functional microsphere (CT@RPS/CS), as an adsorbent, can adsorb heavy metal lead ions and give full play to the antioxidant properties of catechin. CT@RPS/CS would be a potential micron carrier of food functional factors, which may have space to improve and expand the functional characteristics of the loaded functional factors.

## 2 Material and method

### 2.1 Chemicals and reagents

Epicatechin (EC, HPLC ≥ 98%), Epigallocatechin (EGC, HPLC ≥ 98%), Epicatechin gallate (ECG, HPLC ≥ 98%), Epicatechin gallate (EGCG, HPLC ≥ 98%), and a,a-diphenyl-bpicrylhydrazyl (DPPH) were purchased from Sigma Chemical Co., Ltd (St. Louis, MO, USA). Chitosan (degree of deacetylation ≥ 95%, viscosity 100–200 mPa s) was purchased from Aladdin Chemical Reagent Co., Ltd. (Shanghai, China). Milli-Q water was obtained from the Millipore Bedford purification system (MA, USA). Lead acetate (AR, Pb(C_2_H_3_0_2_)_2_·3H_2_0) and acetic acid (purity over 99%) were purchased from Sinopharm Chemical Reagent Co., Ltd.

### 2.2 The preparation of porous chitosan-modified starch preparation

The preparation of the porous chitosan-modified rice starch (RPS/CS) was performed as follows [24]: 1) The preparation of CS solution: 1 g of CS was dissolved in 400 ml of acetic acid solution (2 g/L), and then magnetic stirring and mixing at 20 °C for 30 min. 2) 3 g of RPS was evenly dispersed in CS solution to obtain RPS/CS dispersion with a CS concentration of 25% (w/W); 3) After natural precipitation for 24 h, the supernatant was removed, and the precipitation was freeze-dried (−50 °C, 24 h) to avoid the gelatinization according to reference [25]. And then, the powder sample was heat-treated in an oven at 130 °C for 4 h and cooled to room temperature. The powder (RPS/CS) was collected through a 200-mesh sieve.

### 2.3 The preparation of CT loading RPS/CS

The preparation of CT loading RPS/CS was performed as follows: 1) The preparation of CT solution with the concentration was 2 g/L 2) 1 g NRS, RPS, RPS/CS, and CS were dispersed in 30ml CT solution (2 g/L) and shaken in constant water at 25 °C for 2 h. 3) After centrifugation (6000*g*, 5 min), the supernatant was removed, and the precipitation was freeze-dried (−50 °C, 24 h). Finally, we obtained various CT-loaded composite microspheres labeled CT@NRS, CT@RPS, CT@RPS/CS, and CT@CS.

### 2.4 Adsorption towards CT

1 g NRS, RPS, RPS/CS, and CS was dispersed in 30 ml CT solution (2 g/L) and shook in constant temperature water at 25 °C for various times (2.5, 5, 10, 20, 30, 60, 90, 120, 180,240,360 and 480 min). 2 ml dispersion were centrifuged (6000*g*, 5 min) and used to determine the concentration of CT (the sum of EC, EGC, ECG and EGCG concentrations) in the supernatant by HPLC which refer to GB/T21727-2008, and calculate the CT adsorption capacity *q*_*t*_ (mg/g) of the sample (Eq. (1)):

(1) $$q_{\text{t}}=\frac{\left(c_{0}-c_{t}\right)\text{v}}{\text{m}}$$

In Eq. (1), c_0_ and c_t_ are the initial concentration of CT solution and the concentration at t min (mg/ml), v is the volume of solution (mL), and m is the mass of sample (g). When the balance of adsorption, the concentration and adsorption capacity of the solution are expressed by c_e_ and q_e,_ respectively.

**Figure 1:**
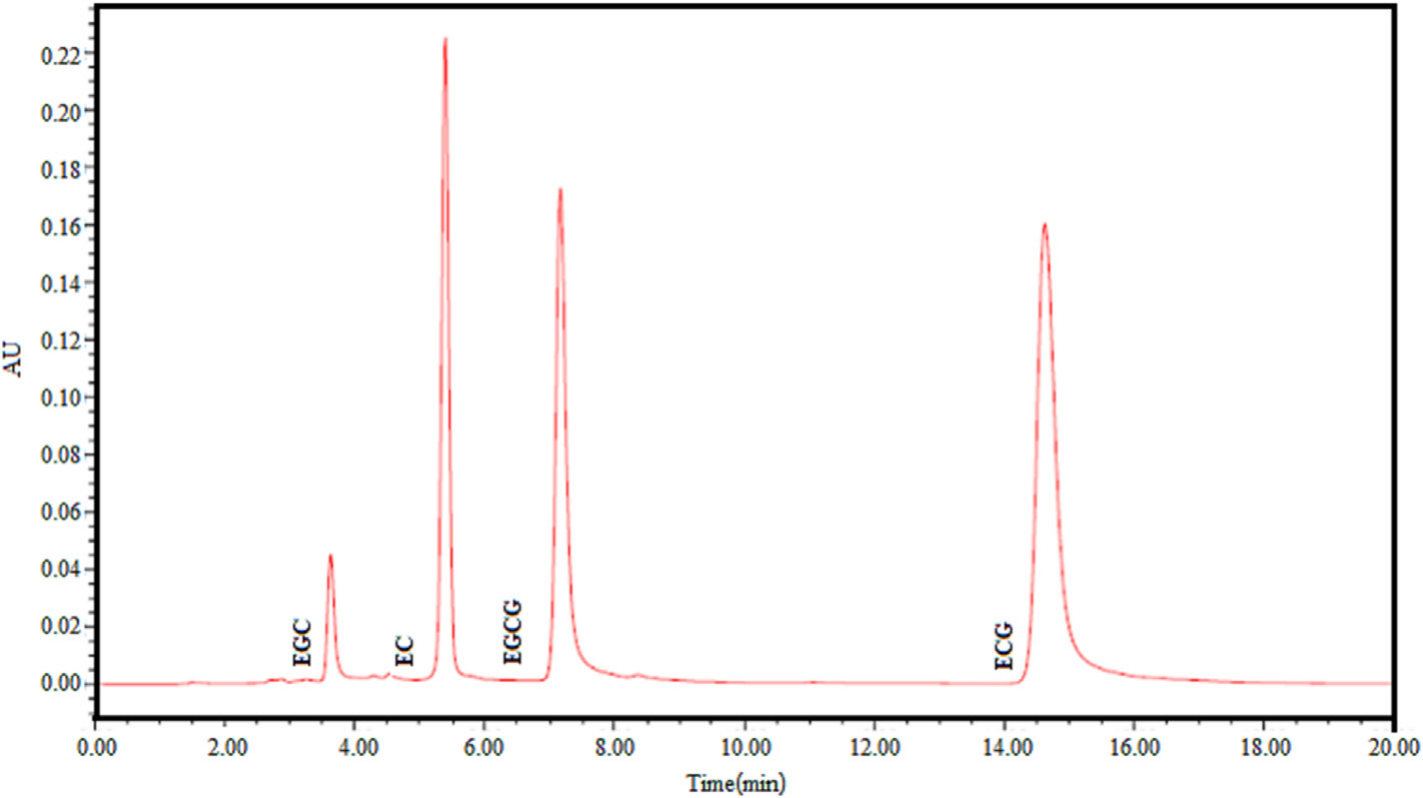
Mixed map of EC, ECG, EGC, and EGCG.

The preparation of CT solution was performed as follows: The standards of EC, EGC, ECG, and EGCG were dissolved in Milli-Q water to prepare the standard solutions of four monomeric catechins, and HPLC was determined as the standard curves of these four standard solutions. The test conditions are as follows: chromatographic column waters X Bridge (4.6 × 25 × 5 μm), The column temperature is 30 °C, and the mobile phase is ultrapure water: acetonitrile: ethyl acetate = 86: 12: 2, flow rate 1.0 ml/min, detection wavelength λ = 280 nm, injection volume 20 μL. The peak time was 19 min, and four groups of standard curves (Table 1) and mixed standard spectra (Figure 1) were obtained.

**Table 1:** Standard curve of EC, ECG, EGC, and EGCG.

	Line relation	R^2^
EC	y = 11884x − 8159.3	0.9995
ECG	y = 28763x − 59670	0.9994
EGC	y = 2392.9x − 5471.4	0.9992
EGCG	y = 19251x − 40693	0.9997

The adsorption mechanism of NRS, RPS, RPS/CS, and Cs on CT, the adsorption process is simulated by pseudo-first-order and pseudo-second-order kinetic models (Eqs. (2) and (3)), respectively.

(2) $$ln\left(q_{e}-q_{e}\right)=\text{ln}q_{e}-k_{1}t$$

(3) $$\frac{\text{t}}{q_{t}}=\frac{1}{k_{2}\times q_{e}^{2}}+\frac{t}{q_{e}}$$

*q*_*e*_ and *q*_*t*_ (mg/g) are the CT adsorption contents at equilibrium and t min, respectively. K_1_ (min^−1^) and K_2_ (g/mg/min) are the equilibrium rate constants of pseudo-first-order and pseudo-second-order equations.

### 2.5 Release experiment of CT

We dissolved 1 g CT@NRS, CT@RPS, CT@RPS/CS, and CT@CS in 30 mL Milli-Q water and shook (120 r/min) in constant temperature water at 25 °C for various times (2.5, 5, 10, 20, 30, 60, 90, 120, 180,240,360 and 480 min). 2 ml dispersion (add 2 ml of the corresponding medium to keep the total volume of the solution unchanged) were centrifuged (6000*g*, 10 min) and used to determine the concentration of EC, EGC, ECG and EGCG in the supernatant were determined by HPLC, and the release q_r_ (mg/g) of CT was calculated:

(4) $$q_{\text{r}}=\frac{c_{t}\text{v}}{\text{m}}$$

In Eq. (4), c_t_ is the concentration of CT solution at t min (mg/ml), v is the volume of solution (mL), and m is the mass of sample (g).

### 2.6 Stability experiment of storage

Take appropriate amount of CT@RPS/CS and CT were placed in sample bottles and stored according to experimental groups. Lightless group: wrap the sample with tin foil and keep it in the medicine cabinet; Drying group: store in a desiccator (silica gel); Natural light group: stored on the test bench; Humidity group: take out the desiccant from the dryer and add an appropriate amount of distilled water to maintain the humidity measuring of 70%. Take the samples at various times (0, 5, 10,15, 20, and 30 d) and determine the concentration of CT (EC, EGC, ECG, and EGCG) in the supernatant by HPLC, and then the CT failure rate p_l_ (%) was calculated:

(5) $$p_{\text{l}}\left(\text{\%}\right)=\frac{c_{0}-c_{n}}{c_{0}}\times 100\text{\%}$$

In Eq. (5), c_0_ represents the initial storage concentration, c_n_ represents the concentration at n days of storage, and n represents the storage day.

### 2.7 Detection of antioxidant stress *in vitro*

#### 2.7.1 Determination of hydroxyl free radical scavenging ability

The hydroxyl free radical (·OH) scavenging capacity was determined by the o-phenanthrene-Fe^2+^ oxidation method. Generally, 1 g CT@NRS, CT@RPS, CT@RPS/CS, and CT@CS were dissolved in 30 mL Milli-Q water and shook (120 r/min) in constant temperature water at 25 °C for 4 h, and then the supernatant was used to carry out the next antioxidant stress experiment *in vitro*.

The 1.0 mL supernatant was taken after centrifugation (6000*g*, 10 min). Tests were performed according to reference [26]. The test sample was mixed with 2.0 mL phosphate buffer (pH 7.4), and then 1.5 ml phenanthroline solution (5 mmol/L), 1.0 ml ferrous sulfate solution (7.5 mmol/L), and 1.0 ml H_2_0_2_ (0.1%) were added and mixed evenly, Milli-Q water was supplied to 10 mL and water bath at 37 °C for 1 h, finally, the absorbance A of the solution were determined at 510 nm. Two blank tubes were labeled undamaged A_0_ and damaged A_1_. A_0_ was not added to H_2_0_2_. A_1_ was added 1.0 mL H_2_0_2_ (0.1%). Take the Vc as the control to calculate the hydroxyl clearance rate (R_·OH_, %):

(6) $$R_{\cdot \text{OH}}\left(\text{\%}\right)=\frac{\text{A}-A_{1}}{A_{0}-A_{1}}\times 100\text{\%}$$

In Eq.(6), A represents the absorbance of the tested solution, A_0_ represents the absorbance of an undamaged A_0_ tube, A_1_ represents the absorbance of a damaged A_1_ tube.

#### 2.7.2 Determination of reducing power

**Figure 2:**
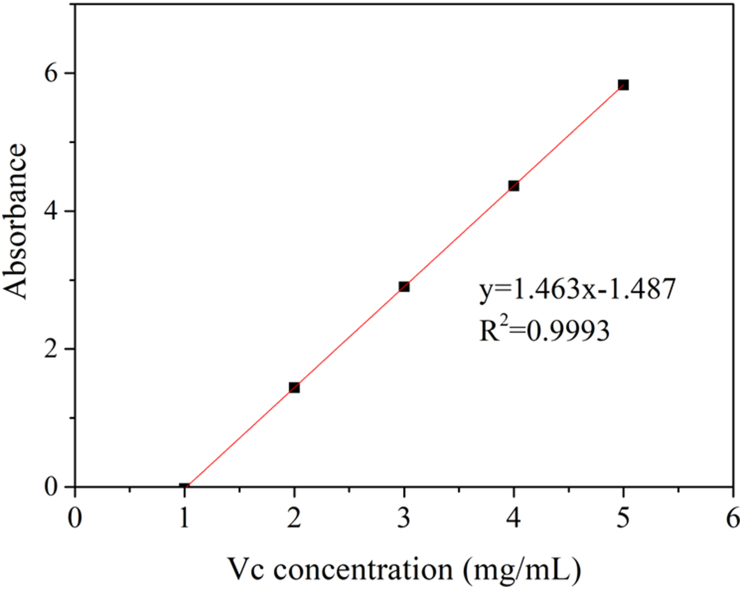
Standard curve of Vc concentration.

The reducing power was determined according to the method [27]. Briefly, 2.5 ml of the supernatant was taken from 1.7.1. 2.5 ml phosphate buffer (pH 6.6) was mixed evenly in the supernatant, and then 2.5 ml potassium ferricyanide solution (1%) was added. The mixture was added and mixed evenly with 2.5 mL trichloroacetic acid solution (10%) after a water bath at 50 °C for 20 min. The 5 ml supernatant after centrifugation (6000*g*, 10 min) and 5 ml Milli-Q water, and 1 mL ferric chloride solution (0.1%) were mixed and used to determine the absorbance of the solution at 700 nm and analyze its reducing power (VC equivalent concentration). Draw the VC concentration standard curve with VC concentration as the abscissa and absorbance as the ordinate (as shown in Figure 2). The fitting standard equation is y = 1.463x − 1.487, R^2^ = 0.9993.

#### 2.7.3 Determination of free radical scavenging ability of DPPH

The scavenging capacity of DPPH was determined according to the method [27]. Generally, take 2.5 ml of the supernatant in 7.1, and 2 ml 2 × 10^−4^mol/L DPPH·ethanol solution was mixed evenly. The mixture was determined the absorbance (A_S_) at 517 nm after avoiding light at 25 °C for 30 min. At the same time, the absorbance A0 (DPPH·ethanol solution) and A1 (sample solution + ethanol solution) were also determined. Calculate the DPPH·clearance rate (RDPPH·, %) (Vc as the control)

(7) $$R_{\cdot \text{DPPH}}\left(\text{\%}\right)=\frac{A_{0}-\left(A_{\text{s}}-A_{1}\right)}{A_{0}}\times 100\text{\%}$$

In Eq.(7), A_0_ represents the absorbance of DPPH·ethanol solution, A_1_ represents the absorbance of sample solution + ethanol solution, and A_S_ represents the absorbance of the test sample.

### 2.8 Digestion experiment *in vitro*

#### 2.8.1 Preparation of simulated gastric juice and simulated intestinal juice

The preparation of simulated gastric juice and intestinal juice according to reference [28], and some improvements were made as follows: 1) Preparation of simulated gastric juice: 0.2g pepsin was dissolved in a certain amount of sterile normal saline (0.5%, w/V), and the pH of the solution was adjusted to 2.0 with hydrochloric acid, and finally the solution was obtained with concentration reach to 3 g/L. 2) Preparation of simulated intestinal juice: 0.04 g trypsin and 0.03 g bile salt were dissolved in 10 ml NaHCO_3_/Na2CO_3_ buffer solution (0.1 mol/L, pH 7). Simulated gastric and intestinal fluid was filtered by a 0.22 μm filter membrane, ready for the next experiment.

#### 2.8.2 Simulate digestion in gastric juice

4 g CT@NRS, CT@RPS, CT@RPS/CS, and CT@CS were taken, and 40 mL simulated gastric juice was mixed, respectively. The mixture was warped with tin foil paper and vibrated in the water bath at 37 °C for various times (0, 30, 60, 120, 240, 360, and 480 min). Then the supernatant was taken after centrifuge (6000*g*, 4 °C, 5 min), and was measured the CT concentration by HPLC.

#### 2.8.3 Simulate digestion in the intestinal juice

4 g CT@NRS, CT@RPS, CT@RPS/CS, and CT@CS were taken, and 40 mL simulated gastric juice was mixed, respectively. The mixture was warped with tin foil paper and vibrated in the water bath at 37 °C for 2 h. Then the precipitate was taken after centrifuge (6000*g*, 4 °C, 10 min) and mixed in 40 mL simulated intestinal juice. The pH of the mixture was adjusted to 7, warped with tin foil paper, and vibrated in the water bath at 37 °C for various times (0, 30, 60, 120, 240, 360, and 480 min). Then the supernatant was taken after centrifuge (6000*g*, 4 °C, 5 min), and was measured the CT concentration by HPLC.

### 2.9 Adsorption characteristics of lead ions by CT@RPS/CS

#### 2.9.1 Correlation between the concentration of Pb^2+^ and the removal rate of Pb^2+^

**Figure 3:**
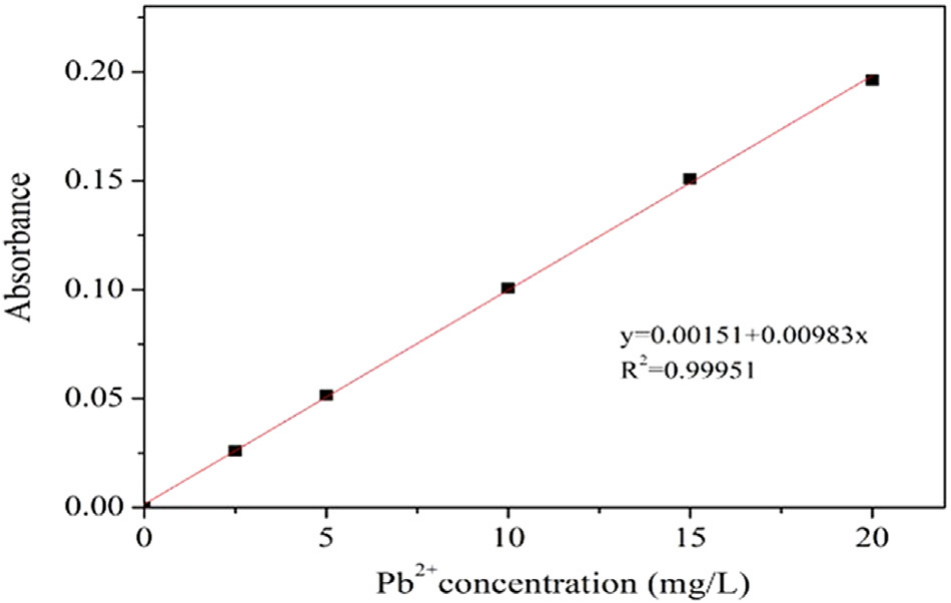
Standard curve of Pb^2+^ concentration.

The lead ion concentration solution was prepared according to the concentration (0, 2.5, 5, 10, 15, and 20 mg/L), the absorbance was determined by atomic absorption spectrometry (AAS), and the Pb^2+^concentration standard curve was drawn out (Figure 3). The fitting equation is y = 0.00983x + 0.00151, R^2^ = 0.99951.

We added 2 g CT@RPS/CS in 50 ml of Pb^2+^ lead acetate solutions of different concentrations (50, 100, 150, 250, 300, 500 μM), respectively. The mixture was shaken in a water bath at 25 °C for 2 h, the supernatant was taken after centrifuge (6000*g*, 4 °C, 10 min), and the concentration of Pb^2+^ was determined by AAS calculated the removal rate (R_Pb_^2+^, %).

(8) $$R_{Pb^{2+}}\left(\text{\%}\right)=\frac{c_{0}-c_{1}}{c_{0}}\times 100\text{\%}$$

In Eq. (8), c_0_ and c_1_ represent the initial concentration of Pb^2+^ and lead ion concentration in the solution after adsorption, respectively.

#### 2.9.2 Effect of the removal rate of Pb^2+^ by adsorbents in solution

The removal rate of Pb^2+^ by adsorbent was 250 μM in lead acetate solution, 2g NRS, CT@NRS, RPS, CT@RPS, RPS/CS, CT@RPS/CS, CS, and CT@CS was added in 50ml 250 μM lead acetate solutions, respectively. The mixture was shaken in a water bath at 25 °C for 2 h, the supernatant was taken after centrifuge (6000*g*, 4 °C, 10 min), and the concentration of Pb^2+^ was determined by formulating 8 and calculating the removal rate (R_Pb_^2+^, %).

#### 2.9.3 Adsorption kinetics of Pb^2+^ by CT @ RPS/CS and FT-IR analysis

We added 2 g CT @ RPS/CS in 50 ml lead acetate solution (250 μM) and vibrated in water bath at 25 °C for various time (0, 2.5, 10, 15, 20, 30, 60, 90, 120, 180, 240, 360 and 480 min). Then took 2 mL of the supernatant and centrifuged (6000*g*, 4 °C, 10 min); finally, the adsorption capacity of Pb^2+^ was determined by AAS and drawled adsorption kinetic curve.

To study the binding form of Pb^2+^ and CT@RPS/CS, the samples were analyzed by FTIR. Tablet was used for potassium bromide, and the scanned range is 400–4000 cm ^−1^.

### 2.10 Data analysis

The experiment was repeated three times in each group. The experimental results were expressed as mean ± standard deviation. The significance analysis was carried out by SPSS13.0 0 statistical soft.

## 3 Result and discussion

### 3.1 Kinetic analysis of CT adsorption by adsorbents

**Figure 4:**
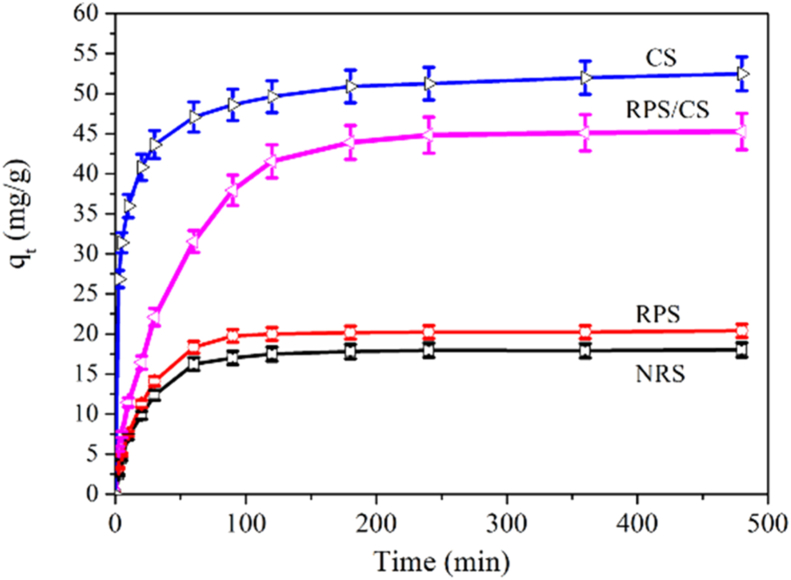
Adsorption kinetics of CT onto samples.

Four adsorption materials (CS, NRS, RPS, and RPS/CS) have an adsorption effect on CT, and there was a noticeable difference among the adsorption characteristics (Figure 4).

The adsorption characteristics of CT are highly similar between NRS and RPS. At the beginning of 20 min, the adsorption rate of NRS and RPS on CT increased linearly and rapidly and reached about 40% of the maximum adsorption capacity; Then, the adsorption rate gradually slowed down after 90 min and tended to balance, reaching their maximum adsorption capacity of 18 mg/g and 21 mg/g respectively. Figure 4 shows that the adsorption capacity of RPS on CT is more significant than NRS at an observed time; it may be because the specific surface of RPS is larger than that of NRS, which is similar to studies [29].

The adsorption characteristics of CS on CT were significantly different from those of NRS and RPS on CT. The adsorption rate of CS on CT increased linearly and sharply within 5 min of the beginning. The adsorption of CS on CT tended to balance after 180 min and reached the maximum adsorption capacity of 50 mg/g. The maximum adsorption capacity of CS on CT was 2.78 times and 2.38 times that of NRS and RPS, respectively. The reason may be that there is mainly a chemically combined between CT and CS, while the primary surface adsorption between starch particles and CT, which is similar to previous studies [24].

RPS/CS for CT tended to balance after 180 min and reached the maximum adsorption capacity of 46 mg/g, 2.56 times, 2.19 times, and 0.92 times of the maximum adsorption of catechin by NRS, RPS, and CS, respectively. These results reflected that the adsorption characteristics of RPS/CS were between (NRS and RPS) and CS on CT.

The adsorption mechanism of NRS, RPS, RPS/CS, and Cs on CT by pseudo-first-order and pseudo-second-order kinetic models, respectively. The results are shown in Table 2. R_2_^2^ in NRS and RPS is slightly lower than R_1_^2^, indicating that the pseudo-first-order model is more effective in stimulating the adsorption of NRS and RPS for CT and reflecting that NRS and RPS on CT belong to physical adsorption, and the control step is mass transfer process [24]. R_2_^2^ in CS is slightly higher than R_1_^2^, indicating that the pseudo-second-order model is more effective in stimulating the adsorption of CS for CT, and reflects the possible adsorption mechanism of CS on CT is mainly chemical adsorption or strong surface complexation [24]. R_2_^2^ and R_1_^2^ are both higher than 0.990 in RPS/CS. They are very closed, indicating that pseudo-first-order and second-order models can effectively simulate the adsorption process of RPS/Cs for CT. The results reflected that the possible adsorption mechanism of RPS/Cs for CT is the coexistence of physical adsorption or chemical adsorption or strong surface complexation. q_e1_ and q_e2_ of RPS/CS were two times higher than those of RPS and NRS, slightly lower than that of CS, indicating that the adsorption capacity of RPS/CS mainly depended on CS [24].

**Table 2:** Pseudo-first-order and second-order equation parameters of CT adsorption onto samples.

Sample	Pseudo-first-order			Pseudo-second-order		
	q_e1_	k_1_	R_1_^2^	q_e1_	k_1_	R_2_^2^
NRS	17.702 ± 0.805^a^	0.957 ± 0.041^a^	0.994	19.331 ± 0.884^a^	0.003 ± 0.00014^a^	0.987
RPS	20.129 ± 1.003^a^	0.957 ± 0.032^b^	0.995	1.964 ± 1.021^a^	0.003 ± 0.00013^a^	0.988
RPS/CS	44.681 ± 2.231^b^	0.977 ± 0.043^c^	0.996	50.695 ± 2.482^b^	0.0005 ± 0.000023^b^	0.992
CS	48.251 ± 2.322^b^	0.809 ± 0.037^c^	0.913	50.659 ± 2.526^b^	0.006 ± 0.00025^b^	0.979

Values (mean ± SD) were calculated using the results from three independent experiments. Values in a column followed by different lowercase letters in superscripts that were significantly different from each other (p < 0.05).

### 3.2 Release characteristics of CT @ carrier in aqueous solution

**Figure 5:**
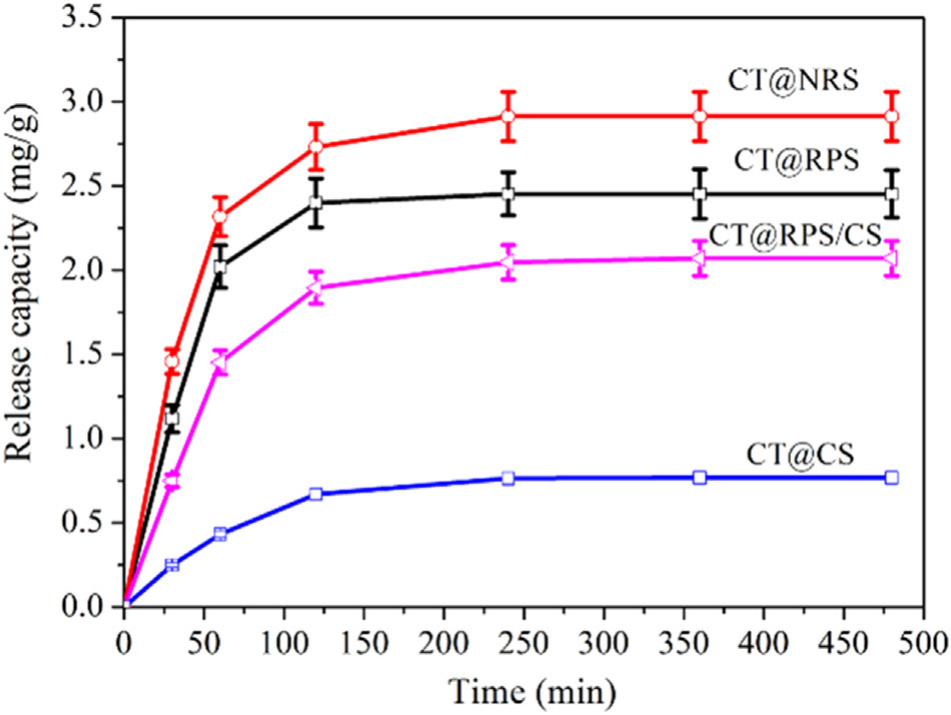
Release curve of CT from samples.

The changing trend of the CT release curve in each sample is the same, the release rate of CT increased rapidly in the initial stage but approached the maximum and stabilized after 4 h, and the order of CT release rate is CT@NRS > CT@RPS > CT@RPS/CS > CT@CS (Figure 5). The driving force of NRS on CT release comes from the concentration difference between the adsorbent surface and CT in the solution. Therefore, the release rate decreases gradually with the increase of release, which is consistent with a previous study [24]. The release mechanism of CT is consistent between CT@RPS and CT@NRS, but because of hinders the diffusion of CT in the micropores of RPS, so CT@RPS releases less CT than CT@NRS, which is consistent with the literature [30]. Due to chemical adsorption or strong surface complexation, this strong intermolecular force makes CT@CS difficult to release CT in an aqueous solution. RPS/CS shows a well-controlled release behavior, and the release amount of RPS/CS for CT is between CT@RPS and CT@CS. The CT release of CT@NRS and CT@RPS reached 2.32 mg/g and 2.02 mg/g after 1 h, respectively. CT@RPS/CS reached 2.05 mg/g after 4 h, which showed a well-controlled release effect, while CT@CS was only 0.76 mg/g after 4 h, which was too slowly released to be applied.

### 3.3 Storage stability characteristics of CT@RPS/CS

**Figure 6:**
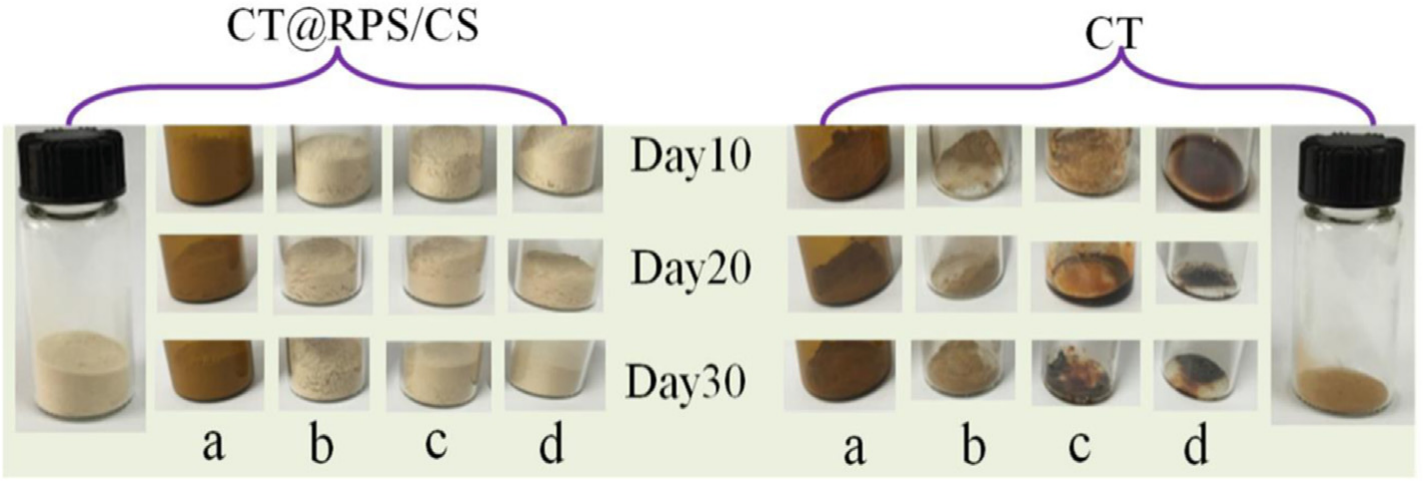
Morphology changes of samples during storage. (a) Arid + Darkness, (b) Arid + Lightness, (c) Darkness + RH 70% and (d) Lightness + RH 70%. The biggest bottle is the morphology of samples before the storage experiment.

Four groups of samples were stored for 30 days, and the changes in the sample's morphology during storage are shown in Figure 6. It has strong water absorption characteristics of CT, but there is no significant impact on CT morphology under dry conditions, whether natural light irradiation or not. However, in the case of humidity 70%, especially in dark conditions, the moisture absorption phenomenon appeared after 10 days, which made the original powder sample gel, and the gel became more obvious as time went on. Similarly, CT@RPS/CS has no obvious moisture absorption in the case of humidity 70% and whether natural light irradiation or not. CT@RPS/CS can better maintain particle morphology in terms of morphology and appearance. These results reflected that RPS/CS could reduce the hygroscopic properties of CT, which may be due to the decrease of hydrophilic properties after the interaction of hydrophilic groups such as phenolic hydroxyl or amino groups with RPS/CS.

Under the same Arid conditions, the results showed that the failure rate of CT@RPS/CS in the natural lightness group was 1.64 times higher than that in the darkness group after 5 days, and the failure rate of CT in the CT group was 2.22 times higher than that in darkness group and compared them have the differences statistically significant (Table 3). There was no significant difference between the failure rate of CT in the CT@RPS/CS group and darkness group during the 10, 15, 20, and 30 days, but in the same stored time, the failure rate of CT in the CT group was higher than that in darkness group and compared them have the differences statistically significant. At the same humidity, the failure rate of CT in both the CS group and CT group increased significantly, especially under the natural lightness, when the failure rate was 5 times that under the same arid conditions. The failure rate of the natural lightness group was higher than that of the darkness group, and that of the humidity (RH70%) group was higher than that of the arid group. Among them, the influence of humidity was more significant than that of light, and the failure rate of the arid and darkness group was the lowest, indicating that arid and darkness were conducive to the storage stability of CT. In conclusion, under the same storage conditions and storage days, the failure rate of the CT group was higher than that of the CT@RPS/CS group. The results show that loading RPS/CS can improve the storage stability of CT, which may be due to the hydrogen bond or electrical action between phenolic hydroxyl groups in CT and CS to protect phenolic hydroxyl groups and inhibit their oxidation failure.

**Table 3:** Effects of storage conditions on loss percentage of CT.

Group		Time(d)				
		5	10	15	20	30
Arid + Darkness (%)	CT@RPS/CS	5.43 ± 0.33^b^	27.44 ± 1.11^ab^	29.11 ± 1.06^b^	32.54 ± 1.36^ab^	35.80 ± 1.54^ab^
	CT	5.42 ± 0.21^b^	30.00 ± 1.13^b^	30.01 ± 1.02^b^	36.00 ± 1.61	38.02 ± 1.98^b^
Arid + Lightness (%)	CT@RPS/CS	8.91 ± 0.43^a^	27.52 ± 1.25^a^	30.63 ± 1.69^a^	34.45 ± 1.18^a^	36.26 ± 2.76^a^
	CT	12.03 ± 0.17	33.02 ± 1.45	37.93 ± 1.84	38.42 ± 1.45	42.92 ± 3.93
Darkness + RH 70% (%)	CT@RPS/CS	23.4 ± 1.91^ac^	27.99 ± 1.13^ab^	33.38 ± 1.64^ac^	35.73 ± 1.93 ^abc^	38.36 ± 1.86 ^abc^
	CT	25.08 ± 1.65^bc^	33.97 ± 1.73^c^	36.24 ± 1.98^c^	37.82 ± 1.41^b^	45.79 ± 1.67^bc^
Lightness + RH 70% (%)	CT@RPS/CS	24.53 ± 1.49^ac^	30.11 ± 1.83^ac^	33.8 ± 1.14^ac^	38.01 ± 1.53^ac^	42.77 ± 1.81^ac^
	CT	31.19 ± 1.81^c^	33.8 ± 1.69	35.32 ± 1.49	46.00 ± 1.37^c^	53.74 ± 1.91^c^

*Note:* a represents p < 0.05 vs. CT, b represents p < 0.05 vs. Lightness, c represents p < 0.05 vs. Arid.

### 3.4 Antioxidant capacity of CT@carrier in water

#### 3.4.1 Hydroxyl radical scavenging capacity

The results of the scavenging capacity of hydroxyl radicals in water are in Table 4. When the concentration of loaded CT increased from 0.5 to 5 mg/ml, their hydroxyl radical scavenging capacity gradually increased. This is consistent with the release behavior of CT @ carrier in an aqueous solution in Figure 5. When the loaded CT concentration reaches 10 mg/ml, the removal rate of hydroxyl radical reaches 100%, indicating that they all have suitable hydroxyl radical removal ability.

**Table 4:** Scavenging capacity of hydroxyl radicals of samples in water.

c (mg/mL)	R_•OH_ (%)			
	CT@NRS	CT@RPS	CT@RPS/CS	CT@CS
10	100	100	100	100
5	92.36	92.11	91.87	90.75
3	81.21	80.33	80.16	78.21
1	71.23	70.16	67.13	55.22
0.5	44.82	44.61	21.37	20.12

*Note:* c represents concentration, R_•OH_(%) represents the scavenging capacity of hydroxyl radicals.

#### 3.4.2 Reducing power

The results of the scavenging capacity of reducing power in water are in Table 5. The change of reduction capacity of the sample in water is consistent with its removal capacity of hydroxyl radical. The reduction ability of CT@NRS and CT@RPS is higher than CT@CS, and the reduced ability of CT@RPS/CS is between CT@NRS and CT@RPS.

**Table 5:** Reducing capacity of samples in water.

c (mg/mL)	c (Vc)			
	CT@NRS	CT@RPS	CT@RPS/CS	CT@CS
6.67	1.91	1.88	1.63	1.39
0.67	1.02	0.97	0.84	0.71
0.33	0.54	0.51	0.41	0.34

*Note:* c represents concentration, and c (Vc) represents the equivalent concentration of Vc.

#### 3.4.3 The free radical scavenging ability of DPPH

The results of the scavenging capacity of reducing power in water are in Table 6. The results showed that the scavenging ability of CT @ carrier to DPPH·in water was like that to hydroxyl radical. It is worth noting that when the concentration of CT@RPS/CS is 5 mg/ml, the clearance rate of DPPH·reaches 100%, indicating that it has good DPPH·clearance ability.

**Table 6:** DPPH free radical scavenging capacity of samples in water.

c (mg/mL)	R_DPPH•_ (%)			
	CT@NRS	CT@RPS	CT@RPS/CS	CT@CS
5	100	100	100	100
3	96.34	95.15	94.12	93.77
1	86.37	85.23	74.12	67.72
0.5	81.47	80.17	53.98	41.71
0.2	46.72	44.17	28.49	22.46

### 3.5 Simulate digestion in gastric juice

**Figure 7:**
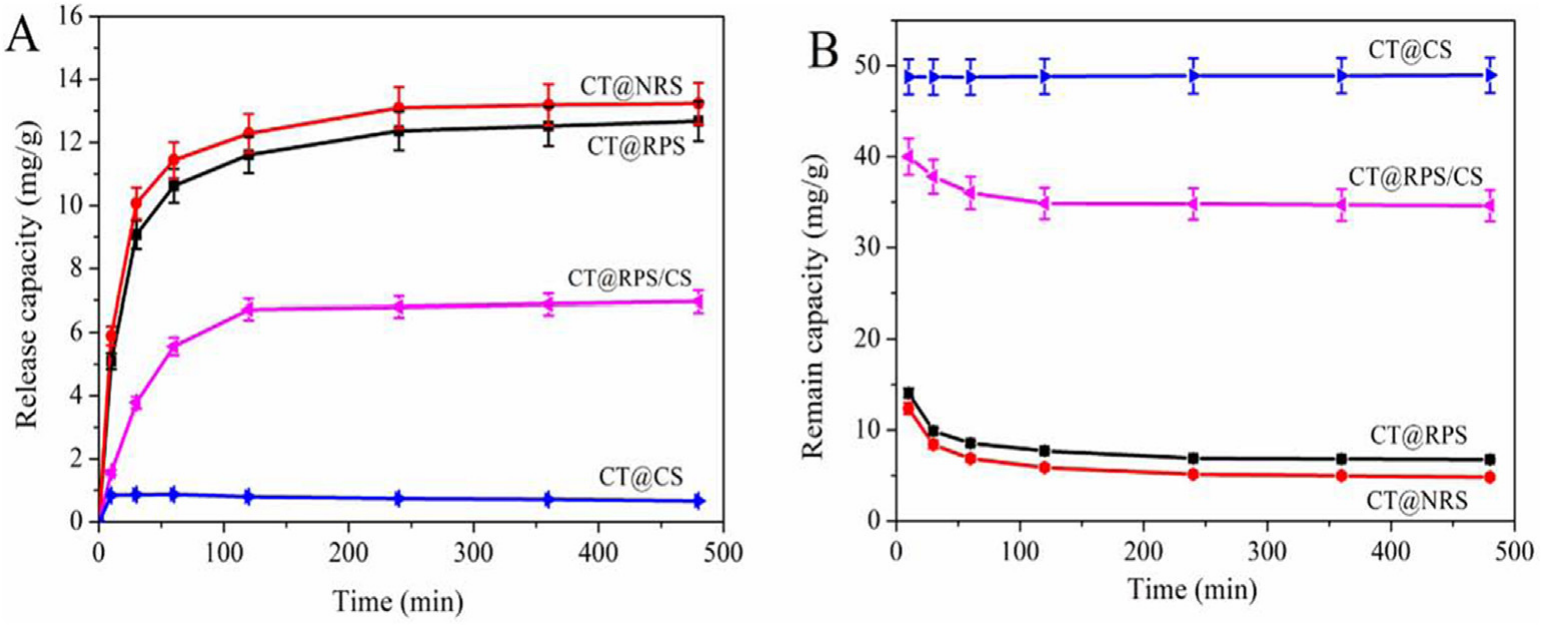
Release (A) and remain (B) curves of CT for samples in simulated gastric juice *in vitro*.

The release curve (Figure 7A) and remain curve (Figure 7B) of CT in simulated gastric juice *in vitro*. The changing trend of the release curve and the remaining curve is consistent. This release characteristic showed that CT@NRS, CT@RPS, and CT@RPS/CS have well-controlled CT release performance in simulated gastric juice *in vitro*. However, the release equilibrium of the CT@CS was reached rapidly within 10 min, and this release characteristic showed that it did not have a well-controlled release performance. It may be due to the binding mode between CT and CS being chemical adsorption or strong surface complexation. The remaining curve of CT on the carrier (Figure 7b) shows that CT release in simulated gastric juice *in vitro* after 8 h, The CT remaining amounts of CT@NRS, CT@RPS, CT@RPS/CS, and CT@CS were 4.81, 6.75, 34.60, and 48.96 mg/g, respectively. The remaining CT in CT@NRS and CT@RPS is relatively small (Figure 7B), which is not enough to maintain its biological activity and sustainable release ability in the intestine. Although the remaining amount of CT was as high as 48.96 mg/g, the release capacity of CT was too poor. It reached the maximum release (0.872 mg/g) and became stable in simulated gastric juice *in vitro* after 10 min (Figure 7A). It should be noted that CT@RPS/CS still retains enough CT to maintain its bioactivity and sustainable release in the intestine after simulating gastric juice *in vitro*.

### 3.6 Simulate digestion in the intestinal juice

**Figure 8:**
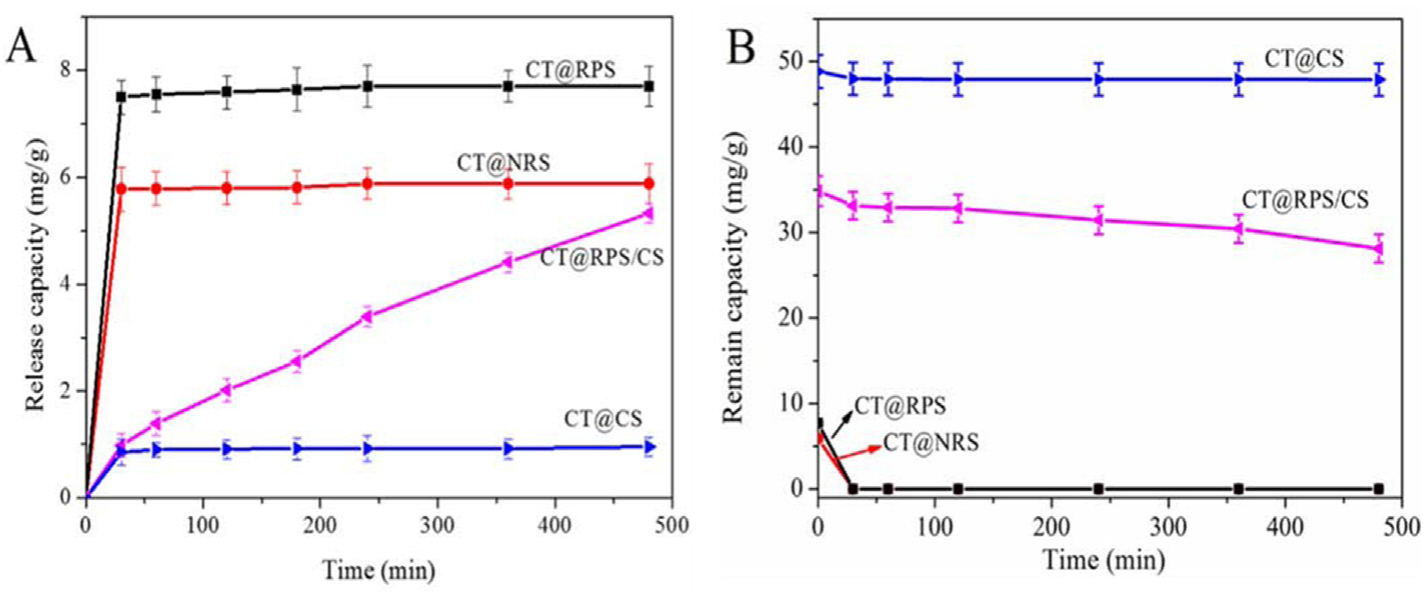
Release (A) and remain (B) curves of CT for samples in simulated intestinal juice *in vitro*.

The release curve (Figure 8A) and remaining curve (Figure 8B) of CT in simulated intestinal juice *in vitro*. Figure 8A showed that the CT release reached the highest and remained stable after 30 min when the CT@NRS and CT@RPS in simulated intestinal fluid, and the retention was close to 0 (Figure 8B). The results reflected that the controlled release ability of CT@NRS and CT@RPS for CT can only be maintained for 30 min in simulated gastric juice, and then the CT has no biological activity in CT@NRS and CT@RPS. The CT release of CT@CS reached 0.85 mg/g after 30 min and remained stable. The release amount was negligible, which reflected that CT@CS is not a well-controlled release characteristic. The CT release amount of CT@RPS/CS was linear with time, indicating that CT@RPS/CS has good stable and sustainable release ability in simulated intestinal fluid *in vitro*. This characteristic is beneficial to maintaining its biological activity and concentration in the intestine.

### 3.7 The removal rate of CT@RPS/CS for Pb^2+^

**Figure 9:**
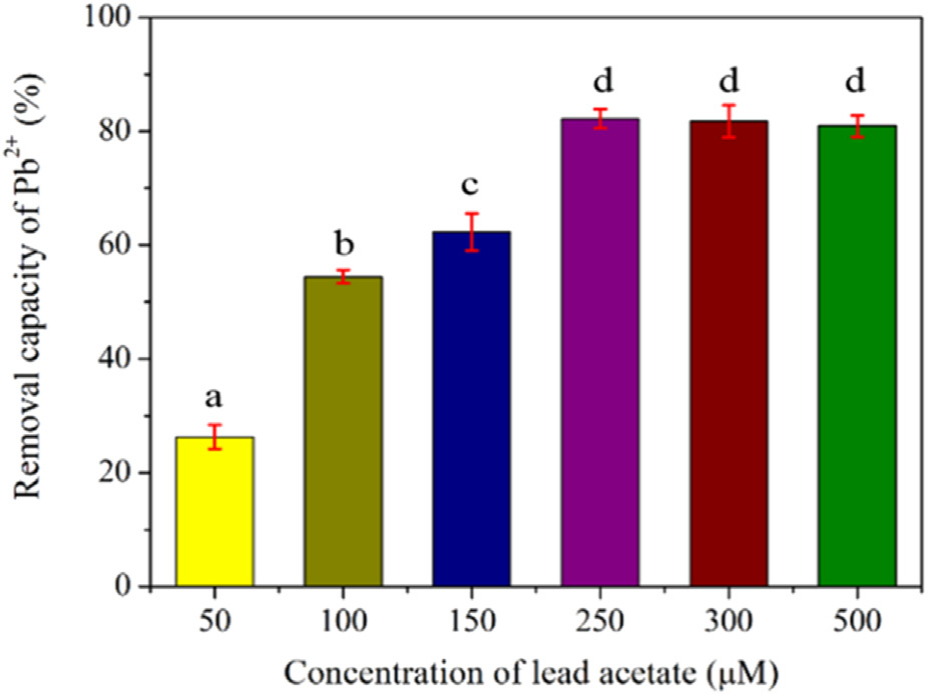
Relationship between removal capacity of CT@RPS/CS towards Pb^2+^ and its concentration. (T = 25 °C, m/V = 1/25 g/mL).

The removal rate of CT@RPS/CS for Pb^2+^ increased with Pb^2+^ concentration (Figure 9). The maximum removal rate of CT@RPS/CS for Pb^2+^ is 82.2% when the concentration of Pb^2+^ is 250 μM. Subsequently, the removal rate of CT@RPS/CS for Pb^2+^ was not increased with the increase of Pb^2+^ concentration. It may be reached adsorbed saturation of CT@RPS/CS for Pb^2+^. According to the experimental results, 250 μM Pb^2+^ solution was selected for subsequent experiments.

**Figure 10:**
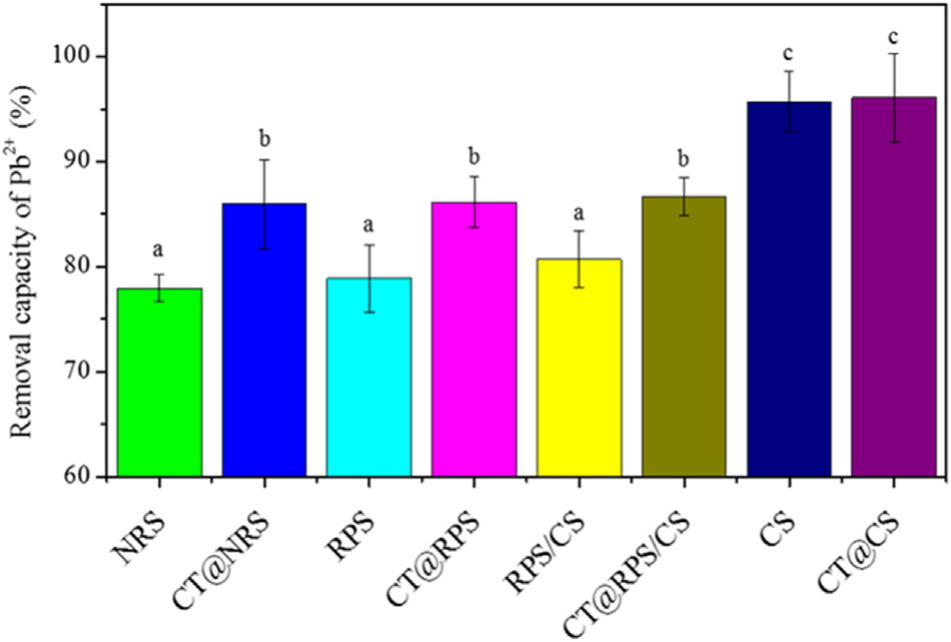
Effects of absorbents on removal capacity of Pb^2+^. *Note:* T = 25 °C, t = 2 h, m/V = 1/25 g/mL, c (Pb^2+^) = 250 μM. a, b and c represent statistical differences in intragroup comparisons, respectively.

To further investigate each component's contribution in CT@RPS/CS to the adsorption of Pb^2+^ in this experiment, the removal rate of Pb^2+^ in 250 μM lead nitrate solution was assessed, as shown in Figure 10. The removal capacity of the four adsorption carriers for Pb^2+^ was CS > RPS/CS > RPS > NRS (Figure 10). The ability of RPS/CS to adsorbed Pb^2+^ is enhanced after CS modifies RPS. At the same time, the removal rates of Pb^2+^ was increased by 0.38%, 7.41%, 9.18%, and 10.27% comparing CS, RPS/CS, RPS and NRS, CT@CS, CT@RPS/CS, CT@RPS, and CT@NRS respectively, indicating that CT has a significant synergistic effect on Pb^2+^ removal, which related to the fact that the phenolic hydroxyl or amino groups of CT are easy to complex with Pb^2+^ and produce chemical adsorption.

### 3.8 Adsorption kinetic analysis of Pb^2+^ by CT@ RPS/CSN and FT-IR analysis

**Figure 11:**
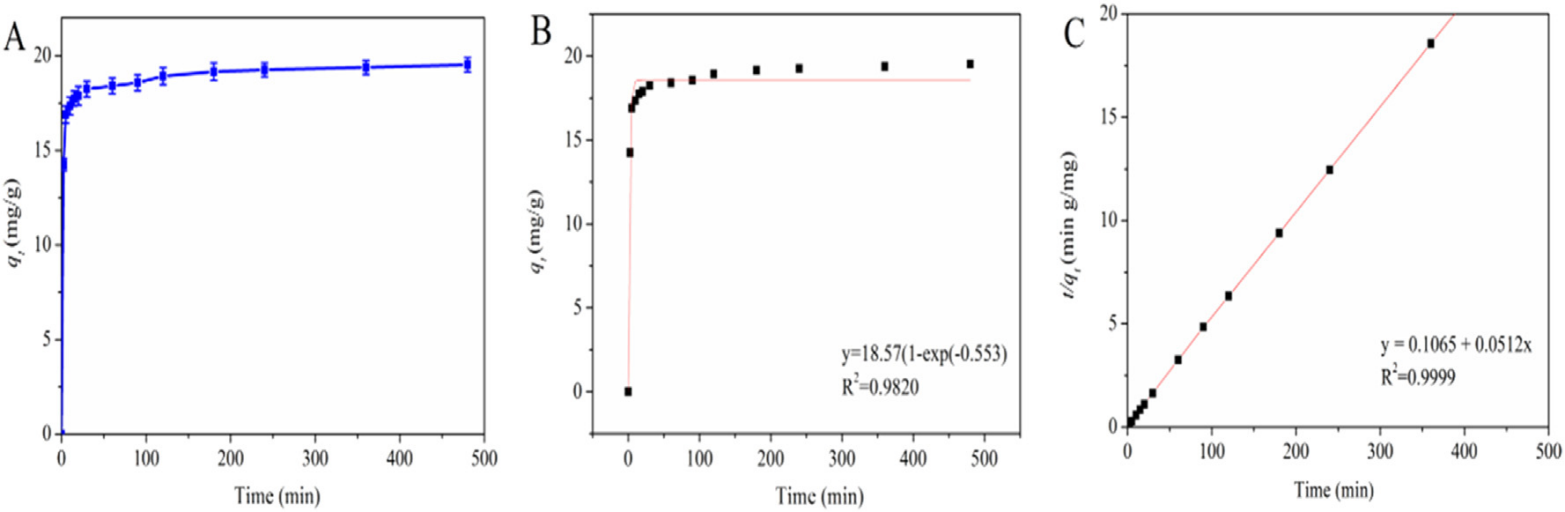
Adsorption kinetics of Pb^2+^ onto CT@RPS/CS. (A) Relationship between Pb^2+^ adsorption and time; (B) Pseudo-first-order model and (C) Pseudo-second-order model. *Note:* (T = 25 °C, m/V = 1/25 g/mL, c (Pb^2+^) = 250 μM).

In this experiment, pseudo-first-order and second-order kinetic simulations were carried out for the adsorption process of CT@RPS/CS and Pb^2+^ (Figure 11). The adsorption rate of CT@RPS/CS for Pb^2+^ is high-speed and reached 17.35 mg/g after 10 min, and then the adsorption capacity tended to be stable (the maximum adsorption capacity is 19.53 mg/g) (Figure 11A). The simulation results of pseudo-first-order kinetics (R^2^ = 0.9820, q_e1_ = 18.57 mg/g) (Figure 11B) and pseudo-second-order kinetics (R^2^ = 0.9999, q_e1_ = 19.53 mg/g) (Figure 11C) suggest that the pseudo-second-order kinetic model can more effectively simulate the adsorption process of Pb^2+^ by CT@RPS/CS. its actual maximum adsorption capacity (19.53 mg/g) is consistent with the pseudo-second-order kinetic model. There are physical adsorption and chemical adsorption of Pb^2+^in the CT@ RPS/CS adsorbing the Pb^2+^, but mainly chemical adsorption (such as chemical complexation between Pb^2+^ and CT.

**Figure 12:**
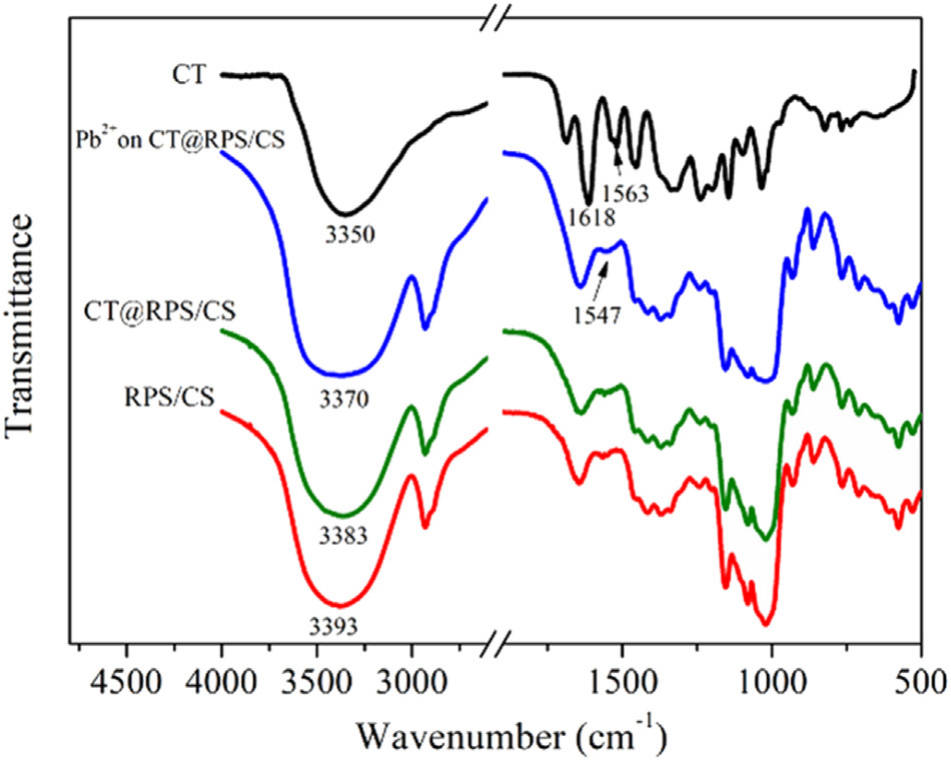
FTIR spectra of samples.

To confirm that the adsorption of Pb^2+^ onto CT@RPS/CS is mainly chemical, the samples were analyzed by FTIR in this study, and the results are shown in Figure 12. The characteristic absorption peaks of CT appear at 3350 and 1618–1530 cm^−1^, representing the stretching vibration of O–H and the skeleton vibration ring of benzene, respectively [31]. When the RPS/CS adsorbed CT, the stretching vibration peak of O–H moved to a low wave (3393 → 3383 cm^−1^), which was attributed to the formation of the hydrogen bond. When the CT@RPS/CS adsorbed Pb^2+^, the stretching vibration peak of O–H moved to a low wave (3383 → 3370 cm^−1^). CT's N– H bending vibration absorption (two bands) shifted to a low wave (1563 → 1547 cm^−1^). This information confirmed the chemical complexation between Pb^2+^ and CT @ RPS/CS.

## 4 Conclusion

The adsorption kinetics study of functional microspheres, confirming the mechanism of the adsorption of RPS/CS to CT was physical adsorption, chemical adsorption, or strong surface complexation, and the adsorption capacity of RPS/CS to CT mainly depended on CS content, which could maintain the particle morphology better. CT@RPS/CS had well-sustained release characteristics with a moderate CT release rate and a sustained-release capacity in an aqueous solution. Its ability to resist oxidation was positively related to the amount of CT released in water. CT has a synergistic effect on Pb^2+^ removal, and CT@RPS/CS significantly enhanced Pb^2+^ removal ability relative to RPS/CS. The results also confirmed the physical adsorption and chemisorption of Pb^2+^ in the adsorption process of CT@ RPS/CS. However, chemical adsorption (chemical complexation between Pb^2+^ and CT) was dominant.

## Declarations

### Author contribution statement

Suwei Jiang: Conceived and designed the experiments; Analyzed and interpreted the data; Contributed reagents, materials, analysis tools or data; Wrote the paper.

Hailiang Hu: Performed the experiments; Analyzed and interpreted the data; Wrote the paper.

### Funding statement

This work was supported by the Project of Anhui Province (2019-03a06020031).

### Data availability statement

Data will be made available on request.

### Declaration of interests statement

The authors declare no conflict of interest.

### Additional information

No additional information is available for this paper.
